# Human Platelet Lysate for Good Manufacturing Practice-Compliant Cell Production

**DOI:** 10.3390/ijms22105178

**Published:** 2021-05-13

**Authors:** Michaela Oeller, Sandra Laner-Plamberger, Linda Krisch, Eva Rohde, Dirk Strunk, Katharina Schallmoser

**Affiliations:** 1 Department of Transfusion Medicine, Paracelsus Medical University of Salzburg, 5020 Salzburg, Austria; m.oeller@salk.at (M.O.); s.laner-plamberger@salk.at (S.L.-P.); l.krisch@salk.at (L.K.); e.rohde@salk.at (E.R.); 2 Spinal Cord Injury and Tissue Regeneration Center Salzburg, Paracelsus Medical University of Salzburg, 5020 Salzburg, Austria; dirk.strunk@pmu.ac.at; 3 Cell Therapy Institute, Paracelsus Medical University of Salzburg, 5020 Salzburg, Austria; 4 GMP Laboratory, Paracelsus Medical University of Salzburg, 5020 Salzburg, Austria

**Keywords:** regeneration, human platelet lysate (HPL), good manufacturing practice (GMP), cell therapy, platelet concentrates, regenerative medicine

## Abstract

Numerous cell-based therapeutics are currently being tested in clinical trials. Human platelet lysate (HPL) is a valuable alternative to fetal bovine serum as a cell culture medium supplement for a variety of different cell types. HPL as a raw material permits animal serum-free cell propagation with highly efficient stimulation of cell proliferation, enabling humanized manufacturing of cell therapeutics within a reasonable timeframe. Providers of HPL have to consider dedicated quality issues regarding identity, purity, potency, traceability and safety. Release criteria have to be defined, characterizing the suitability of HPL batches for the support of a specific cell culture. Fresh or expired platelet concentrates from healthy blood donors are the starting material for HPL preparation, according to regulatory requirements. Pooling of individual platelet lysate units into one HPL batch can balance donor variation with regard to essential platelet-derived growth factors and cytokines. The increasingly applied pathogen reduction technologies will further increase HPL safety. In this review article, aspects and regulatory requirements of whole blood donation and details of human platelet lysate manufacturing are presented. International guidelines for raw materials are discussed, and defined quality controls, as well as release criteria for safe and GMP-compliant HPL production, are summarized.

## 1. Introduction

Over the last decades, the application of stem and progenitor cell-based therapeutics has become of growing interest for the treatment of hematological and neurological disorders, chronic autoimmune diseases, liver, kidney, lung, cardiovascular and infectious diseases, as well as chronic wounds, bone and cartilage repair and others (www.clinicaltrials.gov; search term ‘stem cells’ accessed on 10 April 2021, and [1]). In many cases, ex vivo expansion of cells from various tissue origins is required for the preparation of potent cell-based medicinal products, but the standardization of cell culture conditions is still in progress [2,3,4]. While fetal bovine serum (FBS) is widely used as a cell culture medium supplement for research and clinical-scale cell expansion [5,6,7], it bears the risk for transmission of known and unknown pathogens as well as for xeno-immunization against bovine antigens [8,9,10,11]. Furthermore, ethical issues associated with the common crude methods of FBS collection [12] and the request of the European Medicine Agency (EMA) to avoid animal-derived raw materials for manufacturing cell therapeutics [13] should be taken into consideration.

The most common alternative to FBS is human platelet lysate (HPL), efficiently used for in vitro expansion of human stromal cells but also various other cell types [14,15,16,17,18,19]. Additionally to the plasma-borne substances albumin, fibrinogen and further coagulation factors, lipids, glucose and electrolytes [20], HPL contains a great diversity of growth factors and other bioactive molecules, released during the lysis process from platelet granules [18]. These ingredients may affect the cultured cells and become part of the cell product, assuming HPL production from platelet concentrates derived from certified manufacturing sites only [21]. As HPL is not used as a therapeutic product itself but as a raw material for manufacturing cell-based medicinal products, it is not mandatory to produce HPL under good manufacturing practice (GMP) conditions [3]. In licensed blood centers, the GMP-compliant manufacturing of standard blood products is well established based on sophisticated quality management systems. Standard operating procedures and deviation notification as well as operative risk management systems are applied, according to international guidelines [22]. At the Department of Transfusion Medicine, Paracelsus Medical University of Salzburg, Austria, HPL is routinely produced from expired gamma-irradiated platelet concentrates, prepared by pooling buffy coats and plasma from healthy blood donors. HPL has been implemented for ex vivo propagation of various types of mesenchymal stromal cells (MSCs) for research purposes and clinical application [7,15,20,23,24,25], and for research-grade expansion of endothelial colony-forming progenitor cells (ECFCs) [17,26]. Xeno-free propagation of human myoprogenitor cells (MPCs) under the aegis of HPL was established to treat patients with stress urinary incontinence at the Department of Urology, University of Zurich, Switzerland. This review summarizes the regulatory requirements, HPL manufacturing steps, defined quality controls and release criteria for GMP-compliant HPL production, discussed with regard to European and United States (U.S.) guidelines.

## 2. Safety of Blood Products

Healthy individuals fulfilling all regulatory requirements for blood donation and the withdrawal of blood by trained staff in a safe and clean environment are prerequisites for the safety of blood products [22]. After physical examination and with a medical history confirming suitability for donation, various blood samples are drawn for screening purposes. Analysis includes ABO and Rhesus blood group typing as well as irregular anti-erythrocyte antibody screening. Serological and nucleic acid screening for blood-borne infectious diseases serve to characterize the blood product and improve safety. Blood centers also benefit from an established digital documentation system for manufacturing details and donor and recipient data. This traceability system is also advantageous for HPL used as raw material for the preparation of cell therapeutics [19].

### 2.1. Donor Eligibility

The donor evaluation process usually starts with answering a written questionnaire. A pre-donation interview conducted by trained medical health care professionals collects information on current health constitution, medication, previous diseases, risk behavior for venereal diseases and recent travel activities. In Europe, approved blood donors are usually between 18–65 years old and weigh at least 50 kg. Good general health condition is considered to include normal arterial blood pressure, heart rate and capillary hemoglobin value in addition to an inconspicuous physical examination [22]. Individuals not fulfilling these requirements are refused from donation.

### 2.2. Donor Blood Group Testing and Antibody Screening

Each blood donation is tested for ABO and Rhesus blood groups and irregular anti-erythrocyte antibodies, using serological and in some cases sequence-specific primer (PCR-SSP) technology. ABO testing is mandatory to confirm blood product compatibility [27], as major incompatibility may induce severe or even lethal haemolytic transfusion reactions [28,29,30,31]. For serological typing, anti-A and anti-B sera are used to detect their target antigens on erythrocytes (forward type). In addition, test red blood cells are applied to detect isoagglutinins in the donor’s sera (reverse type) [32]. Usually, molecular testing by PCR-SSP or direct sequencing is only performed in the case of ambiguous serological results, weak antigens or variants.

### 2.3. Donor Screening for Potential Transfusion-Transmitted Diseases

In accordance with international and national health policies, all blood donors are screened for transfusion-transmitted infectious diseases. Two different methodologies are used: (i) serological immunoassay screening for pathogens or antibodies, such as enzyme- or electrochemiluminescence immunoassays (EIA and ECLIA, respectively), hemagglutination or particle agglutination assays and (ii) nucleic acid amplification technology (NAT) screening for the presence of viral RNA or DNA. In Europe, serological screening for antibodies against human immunodeficiency virus 1/2 (HIV1/2), hepatitis C virus (HCV) and hepatitis B virus (HBV) surface antigens is common standard [22]. In addition, serological screening for antibodies directed against the bacterium Treponema pallidum, the causative agent of syphilis, is mandatory in most countries worldwide [33]. Viral NAT screening parameters usually include HIV1/2, HBV and HCV [33,34,35]. Depending on local conditions, further screening for hepatitis A virus (HAV) [36], parvovirus B19 (PB19) [37,38], West Nile virus (WNV) [39,40], dengue virus (DENV) [41], Zika virus (ZIKV) [42,43] or hepatitis E virus (HEV) [44,45] is required.

## 3. Whole Blood Processing and Production of Platelet Concentrates

Each year approximately 118.5 million blood donations are collected globally [46]. From up to 20% of blood donations platelet concentrates are produced (24 million per year) for substitution of thrombocytopenia in patients. Three standard procedures are in use with respective national preferences. The ‘platelet rich plasma (PRP)’ method is most common in the U.S. and Asia [18,47]. The so-called ‘buffy coat’ method is widely used in Europe. For both, anticoagulated whole blood is the initial material, and the pooling of several donations for one therapeutic platelet concentrate dose is necessary. Alternatively, single donor apheresis platelet concentrates can be prepared in 100% plasma or additional additive solution by a cell separator. Apheresis platelet concentrates are more common in high-income countries, presumably due to the need for well-trained staff and special devices, resulting in higher costs [47].

Approximately 450 mL whole blood of eligible donors is drawn into standardized blood bags for the production of selected blood products, including pooled platelet concentrates. After a resting period of several hours and a first centrifugation step, whole blood is separated into plasma, red blood cells and the buffy coat layer. The latter mainly consists of leukocytes and platelets. To reach the required minimum of >2 × 10^11^ platelets per unit in Europe [22] and >3 × 10^11^ platelets per unit in the U.S. [18], four to five buffy coat units and one associated plasma unit or the corresponding volume of additive solution have to be pooled. After a second soft spin centrifugation step, the supernatant, including platelets suspended in plasma or additive solution, is transferred to the storage bag. To confirm sterility, aliquots are monitored by an automated detection system or tested by NAT for microbial contamination.

For the PRP method, whole blood is centrifuged with soft spin, separating plasma and platelets from red blood cells and leukocytes, respectively. After a second centrifugation step, platelets are resuspended in 40–70 mL of plasma to obtain PRP. A therapeutic platelet dose is obtained by pooling 4–6 individual donor PRP units [48]. As residual leukocytes must not exceed 5 × 10^6^/unit in the U.S. and 1 × 10^6^/unit in Europe, a final inline filtration step is required [18,22]. Platelet concentrates are frequently gamma-irradiated and may be stored for a maximum of five to seven days. After expiry, they may still be used as starting material for HPL [19]. A summary of the European parameters for routine quality control of platelet concentrates is shown in Table 1.

Due to (re-)emerging pathogens, the increased screening for selected pathogens is an economic and time-consuming burden [49] and still cannot guarantee complete safety of blood products [50]. Despite sophisticated pathogen screening technologies, there still exists an inevitable ‘diagnostic window’ for detection of various infectious diseases. Fortunately, various pathogen reduction (PR) strategies are now available to minimize the contamination risk, not only for plasma products, but also for platelet concentrates. This further processing step is not yet mandatory, but frequently performed. The most common PR techniques are based on illumination with ultraviolet light, with or without addition of photoactive chemicals, for inducing nucleic acid damage and inhibiting replication of pathogens [51]. PR significantly reduces contamination with bacteria, enveloped viruses and protozoa, but may be insufficient for spores, non-enveloped viruses and prions. There are contradictory data in the literature concerning the adverse effects of PR on platelet biology and function. The in vivo hemostatic function of PR-treated platelets was reported to be comparable to untreated platelets [52]. In addition, the support of in vitro cell proliferation by HPL prepared from PR-treated platelet concentrates was found to be at least comparable to FBS [53,54,55,56,57]. In contrast, affected platelet metabolism and function and increased storage lesions were observed in platelet concentrates after PR treatment [58,59]. Consequently, efficacy and potency of HPL produced from PR-treated platelet concentrates may be affected, although safety may be increased. For a final assessment, more data will be required, including a direct comparison of HPL product aliquots prepared from PR-treated vs. untreated platelet concentrates.

## 4. Preparation of HPL

Only platelet concentrates previously released as clinical blood products are included in the HPL manufacturing procedure. For prevention of lethal transfusion-associated graft-versus-host disease, particularly in immunocompromised patients, platelet concentrates are routinely gamma-irradiated at 25 Gray (Gy) without significant effects on platelet function [60]. Freshly prepared or expired platelet concentrates are suitable starting materials for HPL preparation, as demonstrated previously [18,61,62,63]. Platelets contain a wide range of growth factors and other bioactive molecules in their specific granules [18]. Efficient platelet lysis may be induced physically via repeated freeze/thaw cycles [15], sonication [64] or by solvent/detergent (S/D) treatment [65]. In contrast, the release of growth factors and cytokines after CaCl_2_- [66], human- or recombinant thrombin-induced platelet activation [67] may be insufficient, as shown previously [16], and regulatory approval of these substances for GMP-compliant manufacture may be difficult [18].

**Figure 1:**
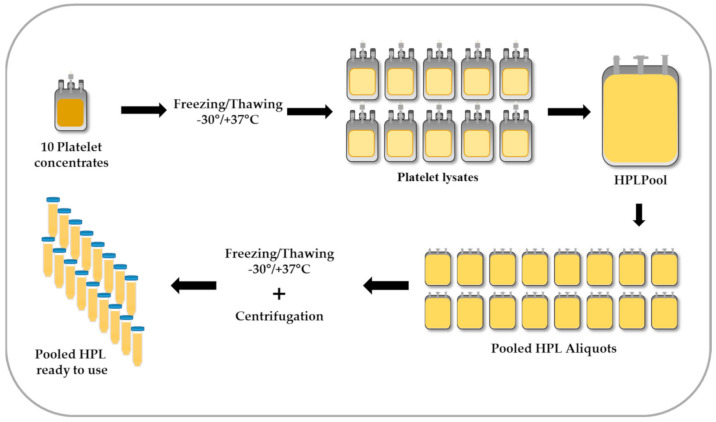
Preparation of pooled human platelet lysate (HPL). Fresh or expired platelet concentrates are exposed to freezing/thawing for platelet lysis. Ten units of platelet lysates are pooled in one bag and further portioned. After a second freeze/thaw step, the bags are centrifuged to deplete the platelet fragments. Final vials of pooled HPL are stored at least at −30 °C until use.

In Figure 1, the procedure of platelet lysis and HPL preparation is depicted. First, buffy coat-derived platelet concentrates are frozen at −30 °C for at least 24 h and thawed at 37 °C to induce lysis. Due to the variable content of serum proteins, growth factors and cytokines in individual platelet concentrates [68], the efficacy of single HPL units may also vary [69]. Notably, in various blood centers HPL pool sizes ranged from a few to more than 100 platelet units [19], whereas low batch-to-batch variations of pooled HPL were observed [20,70,71]. In a recent mathematical approach, the pooling of at least 16 single donor platelet units was calculated as sufficient to improve consistency of various growth factor concentrations and to minimize batch variations of pooled HPL [68]. As a disadvantage of pooling, the potential risk for transfusion-transmitted diseases may increase with the number of donors [3]. Therefore, in Europe a limitation of pool size for raw materials is recommended, unless pathogen inactivation or PR procedures are employed [72].

In our center, up to ten lysed platelet concentrates of various ABO blood groups are pooled in one HPL batch, representing up to 40 blood donations in total. In theory, the combination of blood group O platelets and AB plasma may avoid the exposure of cells to platelet-derived blood group antigens or isoagglutinins in the plasma, respectively [15]. Notably, in a recent international survey, most laboratories stated not to consider ABO blood groups to be important during HPL production [19]. As shown previously, stromal cells do not express AB antigens [73], but endothelial cells do [74], providing the rationale for a specific risk assessment regarding ABO blood group selection for HPL production, depending on the intended use.

After pooling, HPL is sterile-portioned into freezable blood bags. The release of active substances from platelets is increased by a second freeze/thaw cycle. After centrifugation and depletion of platelet fragments, HPL aliquots can be stored in appropriate volume at −30 °C until use for cell culture. The stability of HPL as a raw material has to be assessed for the intended application by the suppliers as well as the users [75], usually in cell proliferation assays and by growth factor analysis. An international survey reported that the recommended maximum shelf life of HPL ranges from 2 years at −30 °C to five years at −80 °C storage temperature [19]. The release of the HPL batch for the manufacturing of cell therapeutics is dependent on quality control results and the evaluation of the defined criteria.

## 5. Quality Control and Release Criteria of HPL

When using HPL or other raw material for the manufacturing of cell-based medicinal products, it may become part of the final product and thus be critical for the safety and purity of the product [76]. The production of cell therapeutics is strictly regulated by international guidelines. For raw materials, only a few quality recommendations have been published by the U.S. Food and Drug Administration (FDA) and the U.S. Pharmacopeia (USP) [76,77]. In 2017, a new chapter for ‘Raw materials for the production of cell-based and gene therapy medicinal products’ of the European Pharmacopeia [72] complemented this framework and included detailed information about sera and serum replacements, as well as platelet lysates. To demonstrate the ‘uniqueness of the raw material’, identity tests should address ‘the molecular structure/composition or other relevant physico-chemical, biological or immunochemical properties’. The raw material should be compared ‘with a defined reference material or a representative batch of the raw material’. However, in the case of sera and serum replacements, the exact composition is difficult to determine. Considering the complex nature of platelet lysates, the consistency and performance of each batch should be verified by defining suitable measures and, at least, the total protein content may be determined. Alternatively, a comparison of albumin content with an appropriate serum reference batch may be applied [72]. The U.S. Pharmacopeia considers the development of functional or performance testing as more suitable, rather than performing identification and characterization via biochemical parameters [77]. General quality requirements include tests for microbiological contamination (bacteria and fungi, endotoxin and mycoplasma), osmolality, pH, and total protein. Hemoglobin content and cell-derived impurities may be tested, where relevant. Finally, the storage conditions, shelf life and proper labeling of the tubes need to be defined [72]. We recommend release criteria for HPL, including negative test results for pathogen screening, bacterial contamination, mycoplasma and endotoxin. Furthermore, biochemical parameters should be available proving the consistency of HPL batches, and the isoagglutinin titer should be ≤1:265. In Table 2, an overview of the recommended quality specifications of pooled HPL in accordance to the European Pharmacopoeia (10th edition) [72] is shown.

### 5.1. Screening for Bacterial and Fungal Contamination

Platelet concentrates carry the greatest risk for bacterial contamination among allogeneic blood products, as they are stored for up to 7 days at room temperature. It was reported that one in 2000–5000 platelet units may be contaminated with bacteria [51]. Sterility of all pharmaceutical products is crucial and should be assured by a validated screening for aerobic and anaerobic bacteria, yeast and fungi. The European Pharmacopoeia suggests direct inoculation and membrane filtration as a reference method [78]. However, due to clouding of the HPL-supplemented cell culture medium and the lack of filterability, this method is not suitable [79]. Alternatively, electrochemical methods based on the detection of bacterial metabolites, such as CO_2_, O_2_ or adenosine triphosphate (ATP), or point-of-issue tests, such as polymerase chain reaction (PCR) assays, flow cytometry or immunoassays for direct measurements, may be used [80].

### 5.2. Testing for Mycoplasma

Mycoplasma are frequently infecting cultured cells, but detection and eradication are challenging [2]. The six most frequently found mycoplasma species were Mycoplasma orale, Mycoplasma arginine, Mycoplasma hyorhinis, Mycoplasma fermentans, Acholaeplasma laidlawii and Mycoplasma salivarium [83]. Mycoplasma may cause changes in cell metabolism and phenotype and therefore may affect biopharmaceutical product quality and safety [84]. Hence, current safety regulations prescribe the testing of cell banks and downstream cell cultures for the presence of viable mycoplasma. The European Pharmacopoeia recommends the culture method for mycoplasma analysis in a final product lot. In addition, the indicator cell culture method may be used for the screening of media to detect species that grow poorly on cell-free media [82]. The culture method is considered as the ‘gold standard’ for the detection of mycoplasma. In brief, test samples are inoculated in parallel on solid agar media and in liquid enrichment media. After incubation, mycoplasma colonies can be identified based on their typical fried egg shape on solid agar. However, conventional mycoplasma culture requires 28 days, delaying the release of a cell product. Furthermore, sensitivity of the culture assay may be affected by inconsistencies in media preparation and quality of the media components [83]. Alternatively, biochemical tests may detect mycoplasma-specific enzyme activity within media, but the sensitivity of approximately 50 colony-forming units (CFU) per mL does not meet current FDA and European Pharmacopeia requirements. According to European guidelines, a properly validated nucleic acid amplification technology (NAT) can replace conventional mycoplasma identification methods [82]. The NAT sensitivity was defined therein to correspond to either 10 (culture-based assays) or 100 CFU/mL (indicator cell culture method) [82]. Hence, mycoplasma detection by NAT has been considered as suitable for HPL quality control.

### 5.3. Endotoxin Test

Endotoxins are toxic breakdown products of Gram-negative bacteria. When inoculated into the blood stream, endotoxins can trigger a severe pyrogenic reaction, activation of the coagulation cascade, hypotension and shock [85]. A maximum threshold of endotoxin concentration has to be defined depending on product type and route of administration. In the European Pharmacopoeia the detection of endotoxins is recommended, using Limulus amebocyte lysate (LAL) obtained from hemolymph of Limulus polyphemus species of horseshoe crabs [81]. Three different techniques can be applied: the gel-clot method, the turbidimetric method and the chromogenic method. The gel-clot method is based on the formation of a clot that remains stable at the bottom of a tube in the presence of endotoxins. The lysate sensitivity is defined as the minimum endotoxin concentration required to induce clotting under standard conditions, expressed in international units (IU). One IU of endotoxin equals one endotoxin unit (EU) [81]. The turbidimetric technique measures gel formation-induced turbidity. Changes in the rate of turbidity and absorbance are proportional to the endotoxin concentration. In the chromogenic method, the natural substrate is replaced by a chromogen-coupled substrate. The chromogenic molecule is released after substrate cleavage by an endotoxin-activated enzyme [81,85]. Recently, the use of recombinant factor C was published as an alternative to the classic LAL method [86]. In the case of HPL, each manufacturer has to define product release criteria, including a threshold of endotoxin concentration. In our center, for example, batches showing an endotoxin concentration below the threshold of 0.5 EU/mL comply with the defined criteria and can be released.

### 5.4. Biochemical Analysis

Composition and potency of HPL are considered to be influenced by platelet concentrate preparation and the mode of lysis, the proportion of plasma and individual donor variations. Platelet concentrates may be prepared either with 100% plasma or a mixture of plasma and additive solution, with respective dilution. Many currently available freeze/thaw HPL preparations are based on 100% plasma formulations and contain a complex mixture of plasma- and platelet-derived proteins [18]. The total protein content ranges from 65 to 85 mg/mL and comprises mostly plasma proteins, such as albumin (35–55 mg/L), fibrinogen (2–4 mg/mL), immunoglobulin (Ig) G (8–12 mg/mL), IgA (0.7–1.3 mg/mL) and IgM (0.3–0.6 mg/mL) [18,21,87]. Some centers routinely perform mechanical or CaCl_2_-induced fibrinogen depletion of HPL to avoid a direct effect of fibrinogen on cell biology and to avoid the need for porcine heparin in the culture medium [66,88,89]. Further essential blood substances, such as lipids, glucose and electrolytes, may serve as biochemical parameters to estimate HPL batch consistency [20,70,71]. For batch identification and comparability, biochemical analysis has to be performed as required [72], including osmolality, pH and total protein. These parameters can directly affect the milieu in the culture medium and are therefore considered critical for the manufacturing of cell therapeutics.

### 5.5. Testing of Isoagglutinin Titer

HPL inherently contains plasma-borne isoagglutinins against blood group antigens. Some cell types, such as endothelial cells, express blood group AB antigens on their surface [74]. High isoagglutinin titers in the culture medium may have effects on endothelial cells in the case of ABO blood group incompatibility. Therefore, the testing of isoagglutinin titers as an additional release criterion might be appropriate. A titer of ≤1:256 was suggested as acceptable threshold [19].

### 5.6. Performance Testing

As HPL is a complex mixture, there is no simple physical, chemical or identity test available for differential batch characterization. To confirm the potency and comparability of HPL batches, the development of performance assays simulating the expected functional attribute is recommended by the U.S. Pharmacopeia [77]. Well-defined performance assays or selected cell proliferation assays with reference cell lines may be used to test lot-to-lot-variability, process reproducibility and finally, product quality.

### 5.7. Platelet-Derived Growth Factors, Cytokines and Chemokines

Besides plasma-borne substances, a variety of specific platelet-derived growth factors, cytokines and chemokines have been detected in HPL, including insulin-like growth factor 1 (IGF-1), platelet-derived growth factor (PDGF)-AA, PDGF-BB, PDGF-AB, transforming growth factor β (TGF-β), brain-derived neurotrophic factor (BDNF), epidermal growth factor (EGF), vascular endothelial growth factor (VEGF), intercellular adhesion molecule 1 (ICAM-1), CC-chemokine ligand 5 (CCL5, RANTES), hepatocyte growth factor (HGF), soluble CD40L, vascular cell adhesion molecule-1 (VCAM-1), basic fibroblast growth factor (bFGF) and others [16,18,20,70,71,90]. Inhibition of PDGF-BB, bFGF and TGF-β by neutralizing antibodies reduced proliferation of stromal cells to a mean of 25%. In contrast, the use of a cocktail of recombinant human (rh) growth factors (rhPDGF-BB, rhbFGF and rhTGF-β1) in the cell culture medium did not sufficiently stimulate cell division [90]. This observation indicates that not single growth factors, but a still undefined mixture of platelet- and plasma derived substances is responsible for the support of cell proliferation.

These growth factors within HPL were found to promote cell proliferation by upregulation of mRNA for the transcription factors myc proto-oncogene protein (cMYC), sex-determining region Y (SRY)-box 2 (SOX2) and Kruppel-like factor 4 (KLF4) in stromal cells compared to FBS-supported cell culture, associated with higher proliferation rates [20]. The precise mechanism is still not completely understood. For quality control, the random analysis of defined growth factors, e.g., PDGF isoforms, may provide surrogate markers for platelet lysis efficiency when comparing individual HPL batches [4].

## 6. Conclusions

Comparing the significance of FBS and HPL for ex vivo expansion of stem and progenitor cells, there are clear advantages of HPL, such as cost effectiveness and highly standardized large-scale manufacturing due to the utilization of expired platelet concentrates. In addition, increased cell proliferation in HPL culture enables sufficient production of safe cell therapeutics within a suitable timeframe. In contrast, the putative risks of FBS, such as xeno-immunization or transmission of bovine pathogens, have to be considered in a risk-based approach. HPL has become a new standard for GMP-compliant cell manufacturing, particularly for cell types where serum-free fully defined media are not yet available. The precise mode of action of this complex mixture of plasma-borne proteins and lipids with platelet-derived growth factors and cytokines still needs to be defined.

## Figures and Tables

**Table 1:** European quality parameters for platelet concentrates (modified from [21,22]). * A minimum of 90% of units tested should meet the required value. Abbreviations: HIV-1/2, human immunodeficiency virus type 1 or type 2; HCV, hepatitis C virus; HBV, hepatitis B virus; SPC, statistical process control.

	Parameters	Range of Specification	Frequency of Testing
**Markers for transfusion-transmissible infections**	Anti-HIV-1/2, anti-HCV, HBV surface antigen; NAT for HIV-1/2, HBV and HCV, syphilis and others as required	Negative by approved test system	All blood donations
**Sterility**	Bacteria and fungi	Negative by approved test system	All units
**Final volume**		>40 mL per 0.6 × 10^11^ platelets	According to SPC
**Biochemical analysis**	pH	>6.4 at the end of storage	According to SPC
**Cell amount**	Platelets *	≥2 × 10^11^/unit	According to SPC
	Residual leukocytes *	<1 × 10^6^/unit	According to SPC

**Table 2:** Quality specifications of pooled human platelet lysate (HPL) recommended in accordance to [72], modified from [21]; * without further pathogen reduction procedure. Abbreviations: EU, endotoxin unit (according to [81]); LAL, limulus amebocyte lysate; PCR, polymerase chain reaction; IAT, indirect antiglobulin test; ELISA, enzyme-linked immunosorbent assay; GF, growth factor.

	Parameter	Range of Specification	Test Method	Frequency of Testing
Pool size	Number of blood donations	10–16 *	-	-
Sterility	Endotoxin	<0.5 EU/mL	LAL endotoxin test [81]	Each batch
	Bacteria and fungi	Negative	Automated microbial detection system, PCR or ELISA	
	Mycoplasma	Negative	Culture [82] or PCR	
Biochemical analysis	pH Osmolality Total protein	According to the range of standard blood values	pH-meter Osmometer Biuret protein assay	Each batch (for 100% plasma HPL)
Immunology	Isoagglutinin titer	Depending on the cell type in culture	IAT	Randomly
Stability	Shelf life	To be validated	Performance testing GF analysis	Randomly
Potency	Cell proliferation	To be validated	Performance testing	Randomly
	Platelet-derived growth factors	To be validated	Multiplex assays, ELISA	

## Data Availability

Data sharing not applicable.

## Abbreviations

ATP	adenosine triphosphate
BDNF	brain-derived neurotrophic factor
bFGF	basic fibroblast growth factor
CCL5/RANTES	CC-chemokine ligand 5
CD40L	CD40 ligand
CFU	colony-forming units
cMYC	myc proto-oncogene protein
DENV	dengue virus
DNA	deoxyribonucleic acid
ECFCs	endothelial colony-forming progenitor cells
EGF	epidermal growth factor
EIA/ECLIA	enzyme- or electro-chemiluminiscence immunoassays
ELISA	enzyme-linked immunosorbent assay
EMA	European Medicine Agency
EU	endotoxin unit
FBS	fetal bovine serum
FDA	U.S. Food and Drug Administration
GF	growth factor
GMP	good manufacturing practice
HAV	hepatitis A virus
HBV	hepatitis B virus
HCV	hepatitis C virus
HEV	hepatitis E virus
HGF	hepatocyte growth factor
HIV-1/2	human immunodeficiency virus type 1 or type 2
HPL	human platelet lysate
IAT	indirect antiglobulin test
ICAM-1	intercellular adhesion molecule 1
IGF-1	insulin-like growth factor 1
IU	international units
KLF4	Kruppel-like factor 4
LAL	limulus amebocyte lysate
MPCs	myoprogenitor cells
MSCs	mesenchymal stromal cells
NAT	nucleic acid amplification technology
PB19	parvovirus B19
PCR	polymerase chain reaction
PCR-SSP	polymerase chain reaction-single specific primer
PDGF	platelet-derived growth factor
PR	pathogen reduction
PRP	platelet rich plasma
rh	recombinant human
RNA	ribonucleic acid
S/D	solvent/detergent
SOX2	sex-determining region Y (SRY)- box 2
SPC	statistical process control
TGF-β	transforming growth factor β
U.S.	United States
USP	U.S. Pharmacopeia
UV	ultraviolet
VCAM-1	vascular cell adhesion molecule-1
VEGF	vascular endothelial growth factor
WNV	West Nile virus
ZIKV	Zika virus
